# CAR-T Cell Therapies: An Overview of Clinical Studies Supporting Their Approved Use against Acute Lymphoblastic Leukemia and Large B-Cell Lymphomas

**DOI:** 10.3390/ijms21113906

**Published:** 2020-05-30

**Authors:** Aamir Ahmad, Shahab Uddin, Martin Steinhoff

**Affiliations:** 1 School of Medicine, University of Alabama at Birmingham, Birmingham, AL 35233, USA; 2 Translational Research Institute, Academic Health System, Hamad Medical Corporation, Doha 3050, Qatar; SKhan34@hamad.qa (S.U.); MSteinhoff@hamad.qa (M.S.); 3 Department of Dermatology and Venereology and Dermatology Institute, Hamad Medical Corporation, Doha 3050, Qatar; 4 Department of Dermatology, Weill Cornell Medicine-Qatar, Qatar Foundation-Education City, Doha 24144, Qatar; 5 College of Medicine, Qatar University, Doha 2713, Qatar; 6 Department of Dermatology, Weill Cornell Medicine, 1300 York Avenue, New York, NY 10065, USA

**Keywords:** CAR-T cell therapy, tisagenlecleucel, axicabtagene ciloleucel, cytokine release syndrome, tocilizumab

## Abstract

Chimeric Antigen Receptor (CAR)-T cell therapy is an exciting development in the field of cancer immunology, wherein immune T-cells from patients are collected, engineered to create ‘CAR’-T cells, and infused back into the same patient. Currently, two CAR-T-cell-based therapies, Tisagenlecleucel and Axicabtagene ciloleucel, are approved by FDA for the treatment of hematological malignancies, acute lymphoblastic leukemia and large B-cell lymphomas. Their approval has been a culmination of several phase I and II clinical studies, which are the subject of discussion in this review article. Over the years, CAR-T cells have evolved to be significantly more persistent in patients’ blood, resulting in a much-improved clinical response and disease remission. This is particularly significant given that the target patient populations of these therapies are those with relapsed and refractory disease who have often progressed on multiple therapies. Despite the promising clinical results, there are still several challenges that need to be addressed. Of particular note are the associated toxicities exemplified by cytokine release syndrome (CRS) and the neurotoxicity. CRS has been addressed by an FDA-approved therapy of its own—tocilizumab. This article focuses on the progress related to CAR-T therapy: the pertinent clinical studies and their major findings, their associated adverse effects, and future perspective.

## 1. Introduction

**Figure 1:**
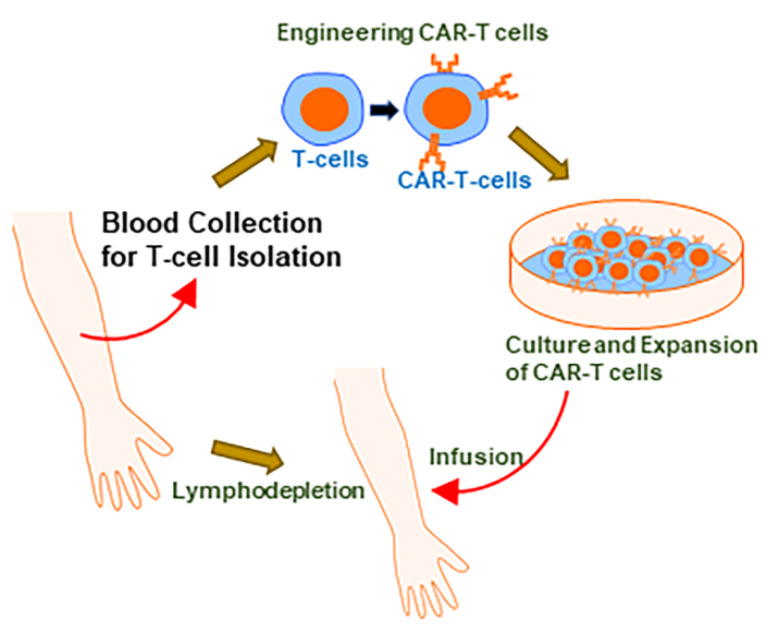
The basics of CAR-T therapy. The first step in CAR-T therapy is the collection of blood from the patient, for the harvesting of T-cells, which are then transduced with the appropriate gene to create ‘CAR-T cells’. These engineered cells are then propagated in the laboratories, to reach a titer that can be effective when infused back in the same individual from whom the T-cells were harvested. Infusion is often preceded by lymphodepletion a-few-days-to-a-week prior.

Chimeric Antigen Receptor (CAR)-T therapy has received a lot of interest in recent years. CAR is a recombinant receptor construct in which an antibody-derived extracellular single-chain variable fragment (scFv) is linked to intracellular T-cell-signaling domains of the T-cell receptor, enabling the redirection of T-cell-mediated cytotoxicity to cancer cells in an HLA-independent manner [1,2]. The scFv, derived from a tumor-specific antibody, endows a new antigen specificity to the reconstructed T-cells [3], allowing for the possible creation of CAR-T cells specific for tumor cells based on the expression of antigens on the surface of tumor cells that can be targeted. The scFv facilitates the binding of CAR-T cells to their targets (tumor cells), while activation of CAR-T cells is made possible by intracellular domains derived from CD3z ITAM domains [3,4]. Within the relative short time of their existence, CAR-Ts have gone through a few generations of evolution. This has been necessitated by modest efficacies and shorter persistence rates in initial studies. While first-generation CARs contained just a CD3z-derived signaling module, the later generations added costimulatory domains [3], with the most-widely evaluated costimulatory domains being CD28 or CD137. These subtle changes in the structure of CAR-T cells have resulted in major clinical advances. Figure 1 depicts the fundamentals of CAR-T therapy. As of now, a few CAR-T therapies have been approved by the United States Food and Drug Administration (US FDA). Tisagenlecleucel (Kymriah) was approved by US FDA for the treatment of acute lymphoblastic leukemia (ALL) and large B-cell lymphomas (Table 1). Axicabtagene ciloleucel (Yescarta) was approved by the US FDA for treatment of certain large B-cell lymphomas (Table 1). In the sections to follow, concise information on the clinical studies that led to their US FDA approval is provided.

## 2. Tisagenlecleucel

Tisagenlecleucel, also known by its trade name, Kymriah, has been approved by US FDA for the treatment of B-cell precursor ALL that has progressed in patients under 25 years of age. This drug is also approved for use in adults with diffuse large B-cell lymphomas (DLBCLs) not otherwise specified, high-grade B-cell lymphomas, or the DLBCLs arising from follicular lymphoma that has progressed after two or more lines of systemic therapy. 

Tisagenlecleucel was initially known as CTL019 during its development at the University of Pennsylvania [5]. CTL019 was developed as an anti-CD19 CAR that included a CD137 signaling domain [6]. CD137, also known as tumor necrosis factor receptor superfamily member 9 (TNFRSF9), 4-1BB, and induced by lymphocyte activation (ILA), is a member of tumor necrosis factor (TNF) receptor family [7]. The primary objective at that time was to be able to engineer CARs that could persist and be effective, prompting the creation of a series of CARs with CD28 and/or CD137 domains. The study [6] revealed that CD137 expressing CARs could survive for at least 6 months in mice with tumor xenografts and, moreover, a complete elimination of primary leukemia xenografts was reported, which was truly remarkable. CD137 influences T-cell proliferation/survival and also protects against apoptosis, all of which might play a role in affording protection to CD137 expressing CARs in circulation.

### 2.1. Tisagenlecleucel: Early Clinical Trial in Adulthood CLL

Among the early challenges during the development of CAR-T therapy was to ensure the persistence of CAR-Ts in the subjects. Subsequent to preliminary design, cloning, and preclinical studies, a phase I study was performed to study the persistence of CAR-Ts in circulation. In one such study [8,9], three males with a history of CLL and multiple therapies and progressions were enrolled in a Phase I trial in 2010 (Table 2). The autologous T-cells were harvested from one patient, prior to the enrollment, in 2009, and subsequent to enrollment in clinical trial, his T-cells were thawed and engineered to express CD19-specific CAR [8]. A total of 3 × 10^8^ T-cells were administered to the patient intravenously. Of these, 5% of the T-cells were engineered ones, i.e., a total of 1.42 × 10^7^ CAR-T cells. An extremely good tumor response was reported, and the trial objective was met with high levels of CAR-T cells found in the blood and bone marrow for six months [8]. The other two patients received engineered CAR-Ts in 2010, after a history of CLL therapies and progressions (Table 2). The CARs were detected in all patients for at least six months, and the toxicities were quite varied. All of the patients reported significant adverse effects of the therapy during two and three weeks post-infusion. However, most of the toxicities were short-termed and reversible. Two of the three patients had complete remission, lasting at least ten months, at the time of reporting, while the third patient had a partial remission that lasted around seven months. It was reasoned that the persisting CAR-Ts consisted of both central and effector memory T-cells, which probably played a role in their persistence for several months.

### 2.2. Tisagenlecleucel: Focus on Pediatric ALL

One of the target patient populations for the CAR-T therapy Tisagenlecleucel is the pediatric patients with ALL [5]. ALL is diagnosed more prominently in children in contrast to the adult. ALL is almost 25% of total cancer diagnoses in children, while in adults, it is just 0.2% of all cancers [5]. Once the persistence of CAR-Ts was established in adult patients with CLL, as discussed in the preceding section, Tisagenlecleucel (CTL019) was then tested in two pediatric patients with ALL [10]. In contrast to the adult CLL patients, who were all males, the pediatric ALL patients were both females. Both had a history of refractory disease. While one patient was administered lymphocyte depletion therapy prior to CAR-T infusion, the other was not (Table 3). Morphologic remission of leukemia with less than 0.01% minimal residual disease was reported for both of the patients approximately a month after the infusions. The two patients had several treatment-related toxicities which largely varied between the two patients (Table 3). The CAR-Ts expanded well in the blood and bone marrow, with levels as high as 1000 times the original engraftment levels detected in the patients. At around three weeks post-infusion, CAR-Ts made up about 1/10^th^ of the circulating T-cells. Unexpectedly, CAR-Ts were even detected in the central nervous systems of both patients. In about a month, complete remission was seen in both patients. In one patient, this persisted >11 months, as of last reported. However, the second patient had a relapse approximately two months post-CAR-T-infusion, with the emergence of CD19-negative blast cells. The therapy was successful in killing CD19-positive cells, as designed, but perhaps the resident CD19-negative precursor cells were inadvertently selected for the emergence of recurrent disease. This observation is in contrast to the above-discussed study [9], with three adult CLL patients, where no such CD19-negative recurrence was reported. Altogether, this trial in pediatric ALL patients provided the first proof for the efficacy of CAR-T therapy in such pediatric population. 

Based on these observations, a phase I/IIA study then evaluated the therapy in a larger cohort of 30 ALL patients that comprised both pediatric patients (*n* = 25) and adults (*n* = 5) [11]. The pediatric population consisted of patients aged between 5 and 22 years (median age = 11) and had 14 males and 11 females. Only three patients had just one ALL relapse, while the rest (i.e., 22) pediatric patients had two or more relapses. The adult patients were aged 26 through 60 (median age = 47), with four males and one female. After the administration of 0.76 × 10^6^–20.6 × 10^6^ CAR-T cells per kilogram of body weight to these patients, a complete remission was seen in 90% (27 of 30) of the patients. Such a response rate, as pointed out by the researchers [11], is exceptional, given the completed remission rate of just 25% for the other FDA-approved therapies for relapsed ALL. With regards to the persistence, CAR-T cells were detectable in the blood of patients for up to 11 months. Of the 27 patients with complete remission at four weeks, seven patients died after they had a relapse between 6 weeks and 8.5 months, post-CAR-T-cells-infusion. Remission was followed up and reported for as long as 24 months, at the time of reporting. Another remarkable finding from this study was a high remission rate (60%) in patients for whom stem cell transplantation had failed. Moreover, among the enrolled patients were three patients who had progressed on blinatumomab. Blinatumomab is a CD19-targeting bispecific antibody approved for relapsed ALL [12]. Of these three patients, two had complete remission, thus suggesting that CD19-targeting CAR-T therapy is promising, even for the patients who have progressed on other CD19-targeting therapies.

Following up on these encouraging results, a multi-center, single-cohort, phase 2 was planned across four continents, exclusively for pediatric patients [13]. This study was named the ELIANA trial [14] and involved patients across 25 hospitals worldwide. The 75 patients who ended up receiving Tisagenlecleucel had a median age of 11 years, with a history of one to eight previous therapies (median of three previous therapies). A majority of them (72 of 75) received lymphodepleting chemotherapy prior to CAR-Ts infusion. The median dose of infused CAR-Ts was 3.1 × 10^6^ per kilogram of body weight (range: 0.2 × 10^6^–5.4 × 10^6^ CAR-Ts per kilogram of body weight). The overall remission rate was 81%, with at least three months of follow-up, and the remissions were observed to be durable, with the six-month relapse-free survival rate and overall survival rates being 80% and 90%, respectively [13]. The relapse-free survival rate was 59% at the year mark, and CAR-Ts were reported to persist in the blood for as long as 20 months (median of 168 days at the data cutoff time). Several transient grade 3, as well as grade 4, adverse events were reported in the patients, with most within the first two months of CAR-Ts’ infusions. Overall, a single Tisagenlecleucel infusion was found to result in highly efficient and durable remission in children and young adults in this global study.

### 2.3. Tisagenlecleucel in Adult B-Cell Lymphoma

With the success of CAR-T therapy in adult CLL and pediatric ALL patients, the focus then turned to evaluating the efficacy of this therapy against adult B-cell lymphomas, more specifically against DLBCL and follicular lymphomas, the number-one and -two most-common non-Hodgkin’s lymphomas, respectively. Enrolled patients representing both of these lymphomas received Tisagenlecleucel infusions during 2014–2016 [15]. In all, 28 patients were infused with CAR-Ts (14 each with DLBCL and follicular lymphoma), and the median age was very similar for the two lymphomas (58 years for DLBCL and 59 years for follicular lymphomas). The gender distribution was very even for follicular lymphoma patients (*n* = 7 males and females), while DLBCL patients had more males (*n* = 11) than females (*n* = 3). Since the eligibility criterion was patients who had progressed after prior therapies, all patients had a history of previous therapies, with the DLBCL patients with a median history of three previous therapies, and the follicular lymphoma patients with a median history of five previous therapies [15]. The median CAR-Ts infused were 5.79 × 10^6^ per kilogram of body weight (range: 3.08 × 10^6^–8.87 × 10^6^). This resulted in complete remission in 6 of 14 patients with DLBCL (43%) and 10 of 14 patients with follicular lymphoma (71%). In all, 57% patients had a complete and sustained response.

A follow-up phase 2 study, JULIET, evaluated the safety and efficacy of Tisagenlecleucel in adult DLBCL patients whose disease had progressed [16]. The inclusion criteria was age > 18 years and a history of at least two prior therapies. Even the patients with DLBCL that had transformed from follicular lymphoma were included. Noteworthy exclusion criteria included previously received CD19-targeted therapies and any indications of central nervous system involvement. A total of 111 DLBCL patients with a median age of 56 years, distributed between a main cohort (*n* = 95) and cohort A (*n* = 16), received CAR-T infusions with median total CAR-T dose of 3.0 × 10^8^ cells (range: 0.1 × 10^8^–6.0 × 10^8^ CAR-T cells). A majority of patients (103 of 111) were administered lymphodepleting chemotherapy prior to CAR-T infusion. Ninety-three patients in the main cohort had >three months of follow-up data, and 37 of these patients (~40%) had a complete response. Interestingly, of these 37 patients with complete response, 16 did not have a complete response at one month post-infusion—12 had a partial response, while the rest (i.e., 4) had a stable disease. However, at the two-months’ time-point, these 16 patients had complete remission. Thirty-five patients were observed to be at complete remission at the three-months’ time-point. Three patients died within a month of infusion, as a result of disease progression. It was observed that patients with remission at three months post-infusion were disease-free until at least six months.

## 3. Axicabtagene Ciloleucel

Axicabtagene ciloleucel, also known by its trade name, Yescarta, has been approved by US FDA for treatment of adult large B-cell lymphoma (including DLBCL not otherwise specified, primary mediastinal large B-cell lymphoma, high-grade B-cell lymphoma, and DLBCL arising from follicular lymphoma) patients with relapsed or refractory disease with a history of two or more lines of systemic therapy. Axicabtagene ciloleucel was the first FDA-approved CD19-tergeting CAR-T therapy for the treatment of patients with large B-cell lymphoma [17]. These lymphomas are a subtype of aggressive non-Hodgkin lymphomas. In a long-term study, it was determined that the complete remission afforded by CAR-Ts in DLBCL patients was for a prolonged time—up to 56 months, as of reporting [18]. 

In contrast to Tisagenlecleucel’s 4-1BB co-stimulatory domain, Axicabtagene ciloleucel has a CD28 co-stimulatory domain. The CAR-Ts with such distinct co-domains can have very distinct phenotypes and behaviors, in vitro, as well as in vivo. These domains can impact the persistence with 4-1BB domains containing CAR-Ts generally believed to proliferate and persist for longer times. CD28 CAR-T cells are known to produce a larger proportion of the effector memory cell subsets, while 4-1BB CAR-T cells are more likely to differentiate into central memory T-cells.

### Axicabtagene Ciloleucel’s Clinical Studies

Axicabtagene ciloleucel, originally called “KTE-C19”, was tested in ZUMA-1 phase I study that evaluated Axicabtagene ciloleucel in a total of seven patients with ages ranging from 29 to 69 years (five males and two females) [19]. Five out of seven patients achieved an objective response, while four of seven achieved a complete response within a month of CAR-T infusion. Three patients were reported with complete remission and detectable levels of CAR-Ts, even after a year of infusion.

Phase 2 of ZUMA study involved 21 centers in the US and one center in Israel [20]. The patient cohorts comprised those with diffuse large B-cell lymphoma (cohort 1; *n* = 77) and primary mediastinal B-cell lymphoma (*n* = 8) or transformed follicular lymphoma (*n* = 16) (cohort 2; total *n* = 24). Thus, a total of 101 patients were included, with a median age of 58 years in cohort 1 and 57 years in cohort 2. Cohort 1 had 50 males and 27 females, while cohort 2 had 18 males and 6 females. The objective response achieved was 82%, while the complete response achieved was 54%. Furthermore, the complete response was 40% at the median follow-up time of 15.4 months [20]. Interestingly, it took up to 15 months for 23 of the patients to achieve complete response, and ongoing durable remissions were observed in patients even after two years of infusions, as of the time of reporting. Moreover, two-year follow-up data from this study [21] revealed an objective response rate of 83%, complete response rate of 58%, and partial response rate of 25%. Furthermore, 10% of patients had a stable disease, while 5% patients had a progressive disease.

A direct comparison of the two FDA-approved CAR-T therapies, Tisagenlecleucel and Axicabtagene ciloleucel, in terms of efficacies in the clinical trials, is provided in Table 4.

## 4. CAR-T-Therapy-Associated Toxicity

A common observation in all the clinical studies involving CAR-T cells is the associated toxicity [22]. Cytokine release syndrome (CRS) remains one of the primary associated adverse effects, while neurologic toxicities have also been widely reported. The toxicity related to CAR-T therapy is graded on a scale of 1–4, based on the symptoms presented [22].

### 4.1. Cytokine Release Syndrome (CRS)

CRS is a very common adverse reaction seen in patients after infusion of CAR-Ts. It is a systemic inflammatory response with often dramatic increases in cytokine levels [23] associated with T-cells’ activation and proliferation [11], which can be fatal if not adequately resolved. The milder manifestations of CRS include high temperatures and myalgias, while the more severe and life-threatening manifestations include vascular leak, hypotension, respiratory/renal insufficiencies, cytopenias, and coagulopathy [11]. CRS has been reported to be very similar to macrophage activation syndrome [10]. Because of the inherent involvement of cytokine production in the activation of T-cells, it is envisioned that perhaps some level of CRS is inevitable. This is consistent with the reports from all clinical studies discussed above. It is also worth mentioning that a majority of CRS instances are self-resolved in the patients. Some patients do require hospitalization due to more severe symptoms, but almost all such instances have been reported to be resolved, often with the administration of non-specific glucocorticoid-based anti-inflammatory therapies. 

### 4.2. Tocilizumab as CRS Therapy

Among the many cytokines associated with CRS, the levels of interleukin-6 (IL-6) have been correlated with CRS severity [24,25]. Consequently, the use of tocilizumab, an antibody against the IL-6 receptor, has been advocated for the management of CRS [26]. In August 2017, tocilizumab was approved by the US FDA for the treatment of CAR-T-therapy-associated CRS [27]. Of note, tocilizumab has been tested in clinical studies associated with both of the FDA-approved CAR-T therapies—Tisagenlecleucel and Axicabtagene ciloleucel.

### 4.3. CAR-T-Therapy-Associated Neurotoxicity

In addition to CRS, the neurotoxicity resulting from CAR-T therapy is well documented [28]. However, its pathophysiology remains poorly understood, resulting in inadequate therapies or preventive measures. CAR-T-cell-related encephalopathy syndrome (CRES) is a common neurotoxic manifestation [29]. CRES can range from mild confusion to fatal cerebral edema. Patients often develop encephalopathy with dysphasia and disorientation. As discussed above, for individual clinicals studies, CAR-T cells have been detected in the central nervous system. Thus, the associated neurotoxicity is not surprising. Clearly, more research is needed to better understand this toxicity.

## 5. Conclusions and Perspective

It is now evident that CAR-T therapy has revolutionized cancer research and treatment. Within a few years, substantial progress has been made, particularly for patients with certain hematological malignancies who had progressed after several lines of therapies and had almost exhausted all potential treatments. Furthermore, there are new indications for continued success of CAR-T therapies. For example, beyond just the expected efficacy of CAR-Ts, the finding that CAR-Ts can be in circulation in the central nervous system [10] provides a hope that the therapy can (a) be effective in suppressing possible central nervous system relapses and (b) potentially be tested against primary central nervous system cancers, as well as central nervous system lymphomas. Furthermore, even though the current approved CAR-T therapies target CD19, there are data to suggest [11] that CAR-T therapy might still be a great therapy for patients who have progressed on other CD19-targeting therapies. Thus, the patients who have progressed on other CD19-targeting therapies still have hope. Among the main challenges associated with CAR-T therapy that still need to be addressed are the several toxicities. These toxicities have led to evaluation of the quality of life in treated patients [30]. Additionally, there is a need to tweak this therapy so as to expand its benefits to solid tumors as well [31,32]. The rapid developments related to CAR-T therapy have excited the scientific community, and hopefully the momentum will be sustained.

## Figures and Tables

**Table 1:** FDA approved CAR-T therapies.

Therapy	Trade Name	Approved for	Patient Population	Approval Date
Tisagenlecleucel	Kymriah	Large B-cell lymphomas	Adult	1 May 2018
		B-cell precursor acute lymphoblastic leukemia	Up to 25 years of age	30 August 2017
Axicabtagene ciloleucel	Yescarta	Large B-cell lymphomas	Adult	18 October 2017

**Table 2:** Early clinical study on expansion and persistence of CAR-Ts in adult Chronic Lymphocytic Leukemia patients.

Gender	Age	History	CAR-T Treatment	Adverse Effects	Outcome	Reference
Male	64	Stage I CLL diagnosis in 1996; Therapies in 2002, 2006 and 2009 with mixed success and intermittent progression	Enrolled in Phase I trial in 2010; Chemotherapy to flush lymphocytes four days prior; 3 × 10^8^ total T-cells (1.46 × 10^5^/kg CAR-T cells) infused i.v.	Acute kidney injury; lymphopenia; tumor lysis syndrome	No evidence of CLL by Day 23, persistence of CAR-Ts at high levels for 6 months; complete remission for 10 months at the time of report	[8,9]
Male	65	CLL diagnosis; Therapies in 2002, 2005, 2006, 2008–2009 with history of progression	Enrolled in Phase I trial in 2010; Chemotherapy to flush lymphocytes; 1.6 × 10^7^/kg CAR-T cells infused i.v.	Transient hypotension	Rapid response. Complete remission for >11 months	[9]
Male	77	CLL diagnosis; Therapies in 2007 and 2009	Enrolled in Phase I trial in 2010; Chemotherapy to flush lymphocytes; 1.0 × 10^7^/kg CAR-T cells infused i.v.	Transient cardiac dysfunction	Partial remission for 7 months	[9]

CLL: Chronic Lymphocytic Leukemia, i.v.: intravenously.

**Table 3:** Clinical study of CAR-Ts in pediatric Acute Lymphoblastic Leukemia patients.

Gender	Age	History	CAR-T Treatment	Adverse Effects	Outcome	Reference
Female	7	ALL diagnosis with history of remissions followed by recurrences	No lymphocyte-depletion therapy prior to CAR-Ts infusion; 1.2 × 10^7^/kg CAR-T cells infused i.v. over 3 consecutive days	Hypotension, Acute vascular leak syndrome, ARDS	Complete remission for >11 months, at the time of reporting	[10]
Female	10	ALL diagnosis with two relapses; history of graft-versus-host disease following transplantation of umbilical-cord blood	Lymphocyte-depletion therapy a week prior to CAR-Ts infusion; 1.4 × 10^6^/kg CAR-T cells infused i.v. in a single dose	Liver toxicity with elevated AST and ALT	Complete remission followed by relapse with CD19-negative blasts	[10]

ALL: Acute Lymphoblastic Leukemia, i.v.: intravenously, ARDS: Acute Respiratory Distress Syndrome, AST: Aspartate AminoTransferase, ALT: Alanine AminoTransferase.

**Table 4:** Reported clinical efficacies of CAR-T therapies.

CAR-T Therapy	Disease	Patients (Number)	Complete Remission (%)	CR (%)	ORR (%)	DFS/R (%)	Reference
Tisagenlecleucel	ALL	30	90	*ND*	*ND*	62	[11]
Tisagenlecleucel	Large B-cell lymphomas	93	38	40	52	64	[16]
Axicabtagene ciloleucel	Large B-cell lymphomas	101	51	54	82	51	[20]

CR: Complete Response, ORR: Objective Response Rate (includes complete and partial response), DFS/R: Disease-Free Survival/Remission, ND: Not determined.
